# The genome sequence of *Moehringia trinervia* (L.) Clairv., 1811 (Caryophyllales: Caryophyllaceae)

**DOI:** 10.12688/wellcomeopenres.26721.1

**Published:** 2026-06-13

**Authors:** Markus Ruhsam, Peter M. Hollingsworth, Royal Botanic Garden Edinburgh Genome Acquisition Lab, Plant Genome Sizing Collective, Darwin Tree of Life Barcoding collective, Wellcome Sanger Institute Tree of Life Management, Samples and Laboratory team, Wellcome Sanger Institute Scientific Operations: Sequencing Operations, Wellcome Sanger Institute Tree of Life Core Informatics team, Tree of Life Core Informatics collective, Darwin Tree of Life Consortium

**Affiliations:** 1 Royal Botanic Garden Edinburgh, Edinburgh, Scotland, UK

**Keywords:** Moehringia trinervia, Three-nerved Sandwort, genome sequence, chromosomal, Caryophyllales

## Abstract

We present a genome assembly of *Moehringia trinervia* (Three-nerved Sandwort; Streptophyta; Magnoliopsida; Caryophyllales; Caryophyllaceae). The assembly consists of two haplotypes with total lengths of 1 244.27 megabases and 1 242.42 megabases. Most of haplotype 1 (98.82%) is scaffolded into 12 chromosomal pseudomolecules. Haplotype 2 was assembled to scaffold level. The mitochondrial sequence has a length of 335.44 kilobases and the plastid genome assembly has a length of 149.69 kilobases. Gene annotation of this assembly on Ensembl identified 21 033 protein-coding genes. This assembly was generated as part of the Darwin Tree of Life project, which produces reference genomes for eukaryotic species found in Britain and Ireland.

## Species taxonomy

Eukaryota; Viridiplantae; Streptophyta; Magnoliopsida; Caryophyllales; Caryophyllaceae; *Moehringia*; *Moehringia trinervia* (L.) Clairv., 1811 (NCBI:txid169227).

## Background

*Moehringia trinervia* is a small annual or short-lived perennial characteristic of open, moist ground in woodlands but also in shaded hedgebanks, along woodland edges and disturbed understory habitats where it typically occurs on moderately nutrient-rich soils ( Stace, 2019). It has a broad distribution across Europe and Asia and is very common in the British Isles, although often overlooked as it is mistaken for the very similar looking chickweed ( *Stellaria media*).

Reproduction in *M. trinervia* is primarily sexual, via small, inconspicuous, insect-pollinated flowers. As a mainly annual species lacking vegetative propagation, three-nerved sandwort is likely to rely on seed production rather than clonal growth for population maintenance ( Stace, 2019). Seeds in the genus *Moehringia* possess lipid-rich appendages (strophioles) that attract ants. Field observations suggest myrmecochory (ant-mediated dispersal) as an effective secondary dispersal mechanism for species in the genus, potentially including *M. trinervia* ( Casazza *et al.*, 2008).

*M. trinervia* has been identified as a bioindicator of heavy metal pollution in forest ecosystems, accumulating elevated concentrations of heavy metals, including lead, cadmium, and zinc, in its tissues when growing on contaminated soils ( Godzik, 1992). The study showed that metal concentrations in the plant reflected levels present in the surrounding environment, making it a suitable indicator for assessing anthropogenic impacts on forest floor vegetation ( Godzik, 1992).

*M. trinervia* is a diploid with 2 *n* = 24 ( Lövkvist & Hultgård, 1999; Stace, 2019). There is a single chromosome count of 2 *n* = 24 from material sampled from the UK ( Henniges *et al.*, 2022).

## Methods

### Sample acquisition, flow cytometry and DNA barcoding

A specimen of *Moehringia trinervia* (specimen ID EDTOL05488, ToLID dcMoeTrin1; Figure 1) was used for genome sequencing. It was collected from Aberlady Nnr, United Kingdom (latitude 56.016, longitude −2.8464) on 2023-04-25. The specimen was collected and identified by Markus Ruhsam. The dcMoeTrin1 specimen was also used for Hi-C and RNA sequencing. The herbarium voucher associated with the sequenced plant is E01358095 and is deposited in the herbarium of RBG Edinburgh (E) [ https://data.rbge.org.uk/herb/E01358095].

**Figure 1.**
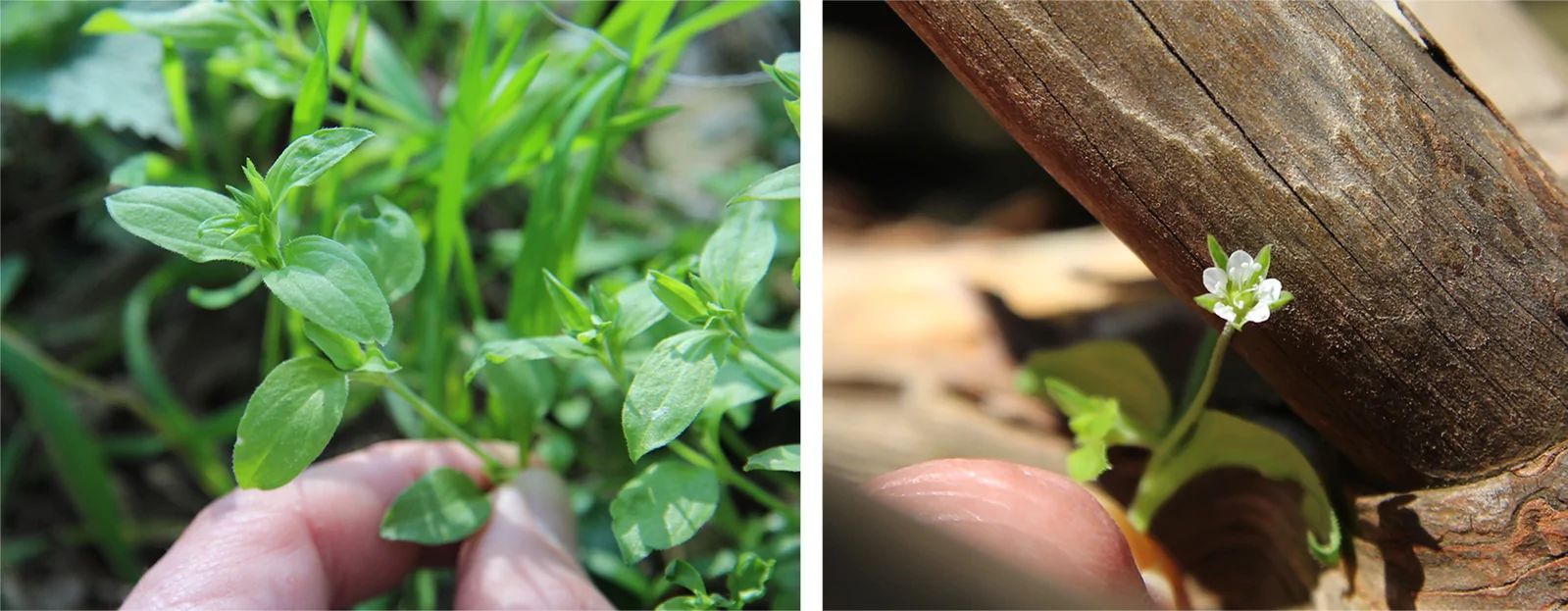
Photographs of the *Moehringia trinervia* (dcMoeTrin1) specimen from which samples were taken for genome sequencing.

The genome size was estimated by flow cytometry following the ‘one-step’ method outlined in Pellicer *et al*. (2021) and using propidium iodide as the fluorochrome. The General Purpose Buffer (GPB) supplemented with 3% PVP was used for isolation of nuclei ( Loureiro *et al.*, 2007), and the internal calibration standard was *Solanum lycopersicum* L. ‘Stupické polní rané’ with an assumed 1C-value of 968 Mb ( Doležel *et al.*, 2007).

The initial identification was verified by an additional DNA barcoding process according to the framework developed by Twyford *et al*. (2024). Part of the plant specimen was preserved in silica gel desiccant ( Chase & Hills, 1991). DNA extracted from the dried plant was amplified by PCR for standard barcode markers, with the amplicons sequenced and compared to public sequence databases including GenBank and the Barcode of Life Database (BOLD) ( Ratnasingham & Hebert, 2007). Following whole genome sequence generation, the relevant DNA barcode region was also used alongside the initial barcoding data for sample tracking at the WSI ( Twyford *et al.*, 2024). The standard operating procedures for Darwin Tree of Life barcoding are available on protocols.io.

### Nucleic acid extraction

Protocols for high molecular weight (HMW) DNA extraction developed at the Wellcome Sanger Institute (WSI) Tree of Life Core Laboratory are available on protocols.io ( Howard *et al.*, 2025). The dcMoeTrin1 sample was weighed and triaged to determine the appropriate extraction protocol. Tissue from the whole plant was homogenised by cryogenic disruption using the Covaris cryoPREP® Automated Dry Pulverizer. HMW DNA was extracted using the Plant Organic Extraction protocol. DNA was sheared into an average fragment size of 12–20 kb following the Megaruptor®3 for LI PacBio protocol. Sheared DNA was purified by automated SPRI (solid-phase reversible immobilisation), using AMPure PB beads (Pacific Biosciences) and the Thermo Fisher KingFisher™ Apex to eliminate shorter fragments and concentrate the DNA. The concentration of the sheared and purified DNA was assessed using a Nanodrop spectrophotometer and Qubit Fluorometer using the Qubit dsDNA High Sensitivity Assay kit. Fragment size distribution was evaluated by running the sample on the FemtoPulse system. For this sample, the final post-shearing DNA had a Qubit concentration of 25.6 ng/μL and a yield of 3 328.00 ng. The 260/280 spectrophotometric ratio was 1.72, and the 260/230 ratio was 2.0. The Genomic Quality Number (GQN) was 8.5.

RNA was extracted from whole plant tissue of dcMoeTrin1 in the Tree of Life Laboratory at the WSI using the RNA Extraction: Automated MagMax™ *mir*Vana protocol. The RNA concentration was assessed using a Nanodrop spectrophotometer and a Qubit Fluorometer using the Qubit RNA Broad-Range Assay kit. Analysis of the integrity of the RNA was done using the Agilent RNA 6000 Pico Kit and Eukaryotic Total RNA assay.

### PacBio HiFi library preparation and sequencing

Library preparation and sequencing were performed at the WSI Scientific Operations core. Libraries were prepared using the SMRTbell Prep Kit 3.0 (Pacific Biosciences) according to the manufacturer’s instructions. The kit includes reagents for end repair/A-tailing, adapter ligation, post-ligation SMRTbell bead clean-up, and nuclease treatment. Size selection and clean-up were performed using diluted AMPure PB beads (Pacific Biosciences). DNA concentration was quantified using a Qubit Fluorometer v4.0 (ThermoFisher Scientific) and the Qubit 1X dsDNA HS assay kit. Final library fragment size was assessed with the Agilent Femto Pulse Automated Pulsed Field CE Instrument (Agilent Technologies) using the gDNA 55 kb BAC analysis kit.

The sample was sequenced on a Revio instrument (Pacific Biosciences). The prepared library was normalised to 2 nM, and 15 μL was used for making complexes. Primers were annealed and polymerases bound to generate circularised complexes, following the manufacturer’s instructions. Complexes were purified using 1.2X SMRTbell beads, then diluted to the Revio loading concentration (200–300 pM) and spiked with a Revio sequencing internal control. The sample was sequenced on a Revio 25 M SMRT cell. The SMRT Link software (Pacific Biosciences), a web-based workflow manager, was used to configure and monitor the run and to carry out primary and secondary data analysis.

### Hi-C

***Sample preparation and crosslinking***

Hi-C data were generated from the whole plant tissue of dcMoeTrin1 using the Arima-HiC v2 kit (Arima Genomics). Tissue was finely ground using the Covaris cryoPREP Dry Pulverizer (Covaris), and then subjected to nuclei isolation. Nuclei were isolated using a modified protocol based on the Qiagen QProteome Cell Compartment Kit (Qiagen), in which only the Lysis and CE2 buffers were used, with QIAshredder spin columns. After isolation, nuclei were fixed using formaldehyde to a final concentration of 2% to crosslink the DNA. The crosslinked DNA was then digested and biotinylated according to the manufacturer’s instructions. A clean-up step was performed with SPRIselect beads before library preparation. DNA concentration was quantified using the Qubit Fluorometer v4.0 (Thermo Fisher Scientific) and the Qubit HS Assay Kit, following the manufacturer’s instructions.

***Hi-C library preparation and sequencing***

Biotinylated DNA constructs were fragmented using a Covaris E220 sonicator and size selected to 400–600 bp using SPRISelect beads. DNA was enriched with Arima-HiC v2 kit Enrichment beads. End repair, A-tailing, and adapter ligation were carried out with the NEBNext Ultra II DNA Library Prep Kit (New England Biolabs), following a modified protocol where library preparation occurs while DNA remains bound to the Enrichment beads. Library amplification was performed using KAPA HiFi HotStart mix and a custom Unique Dual Index (UDI) barcode set (Integrated DNA Technologies). Depending on sample concentration and biotinylation percentage determined at the crosslinking stage, libraries were amplified with 10–16 PCR cycles. Post-PCR clean-up was performed with SPRISelect beads. Libraries were quantified using the AccuClear Ultra High Sensitivity dsDNA Standards Assay Kit (Biotium) and a FLUOstar Omega plate reader (BMG Labtech).

Prior to sequencing, libraries were normalised to 10 ng/μL. Normalised libraries were quantified again to create equimolar and/or weighted 2.8 nM pools. Pool concentrations were checked using the Agilent 4200 TapeStation (Agilent) with High Sensitivity D500 reagents before sequencing. Sequencing was performed using paired-end 150 bp reads on the Illumina NovaSeq X.

### RNA library preparation and sequencing

Libraries were prepared using the NEBNext® Ultra™ II Directional RNA Library Prep Kit for Illumina (New England Biolabs), following the manufacturer’s instructions. Poly(A) mRNA in the total RNA solution was isolated using oligo (dT) beads, converted to cDNA, and uniquely indexed; 14 PCR cycles were performed. Libraries were size-selected to produce fragments between 100–300 bp. Libraries were quantified, normalised, pooled to a final concentration of 2.8 nM, and diluted to 150 pM for loading. Sequencing was carried out on the Illumina NovaSeq X to generate 150-bp paired-end reads.

### Genome assembly

Prior to assembly of the PacBio HiFi reads, a database of *k*-mer counts ( *k* = 31) was generated from the filtered reads using FastK. GenomeScope2 ( Ranallo-Benavidez *et al.*, 2020) was used to analyse the *k*-mer frequency distributions, providing estimates of genome size, heterozygosity, and repeat content.

The HiFi reads were assembled using Hifiasm in Hi-C phasing mode ( Cheng *et al.*, 2021, 2022), producing two haplotypes. Hi-C reads ( Rao *et al.*, 2014) were mapped to the primary contigs using bwa-mem2 ( Vasimuddin *et al.*, 2019). Contigs were further scaffolded with Hi-C data in YaHS ( Zhou *et al.*, 2023), using the --break option for handling potential misassemblies. The scaffolded assemblies were evaluated using Gfastats ( Formenti *et al.*, 2022), BUSCO ( Manni *et al.*, 2021) and MerquryFK ( Rhie *et al.*, 2020). The organelle genomes were assembled using OATK ( Zhou *et al.*, 2025).

### Assembly curation

The assembly was decontaminated using the Assembly Screen for Cobionts and Contaminants ( ASCC) pipeline. TreeVal was used to generate the flat files and maps for use in curation. Manual curation was conducted primarily in PretextView and HiGlass ( Kerpedjiev *et al.*, 2018). Scaffolds were visually inspected and corrected as described by Howe *et al*. (2021). Manual corrections included 12 breaks, 19 joins, and removal of five haplotypic duplications. This reduced the scaffold count by 3.8%. The curation process is described at https://gitlab.com/wtsi-grit/rapid-curation . PretextSnapshot was used to generate a Hi-C contact map of the final assembly.

### Assembly quality assessment

The MerquryFK tool ( Rhie *et al.*, 2020) was run in a Singularity container ( Kurtzer *et al.*, 2017) to evaluate *k*-mer completeness and assembly quality for both haplotypes using the *k*-mer database ( *k* = 31) computed prior to genome assembly. The analysis outputs included assembly QV scores and completeness statistics.

The genome was analysed using the BlobToolKit pipeline, a Nextflow implementation of the earlier Snakemake version ( Challis *et al.*, 2020). The pipeline aligns PacBio reads using minimap2 ( Li, 2018) and SAMtools ( Danecek *et al.*, 2021) to generate coverage tracks. It runs BUSCO ( Manni *et al.*, 2021) using lineages identified by querying NCBI datasets ( O’Leary *et al.*, 2024). For the three domain-level lineages, BUSCO genes are aligned to the UniProt Reference Proteomes database ( Bateman *et al.*, 2023) using DIAMOND blastp ( Buchfink *et al.*, 2021). The genome is divided into chunks based on the density of BUSCO genes from the closest taxonomic lineage, and each chunk is aligned to the UniProt Reference Proteomes database with DIAMOND blastx. Sequences without hits are chunked using seqtk and aligned to the NT database with blastn ( Altschul *et al.*, 1990). The BlobToolKit suite consolidates all outputs into a blobdir for visualisation. The BlobToolKit pipeline was developed using nf-core tooling ( Ewels *et al.*, 2020) and MultiQC ( Ewels *et al.*, 2016), with containerisation through Docker ( Merkel, 2014) and Singularity ( Kurtzer *et al.*, 2017).

## Genome sequence report

### Sequence data

The genome of a specimen of *Moehringia trinervia* was sequenced using Pacific Biosciences single-molecule HiFi long reads, generating 49.19 Gb (gigabases) from 3.91 million reads, which were used to assemble the genome. GenomeScope2.0 analysis estimated the haploid genome size at 1 367.16 Mb, with a heterozygosity of 0.16% and repeat content of 57.54% ( Figure 2). Using flow cytometry, the genome size (1C-value) of the sample was estimated to be 1.42 pg, equivalent to 1 390.00 Mb. These estimates guided expectations for the assembly. Based on the estimated genome size, the sequencing data provided approximately 35× coverage. Hi-C sequencing produced 208.42 Gb from 690.14 million reads, which were used to scaffold the assembly. RNA sequencing data were also generated and are available in public sequence repositories. Table 1 summarises the specimen and sequencing details.

**Figure 2.**
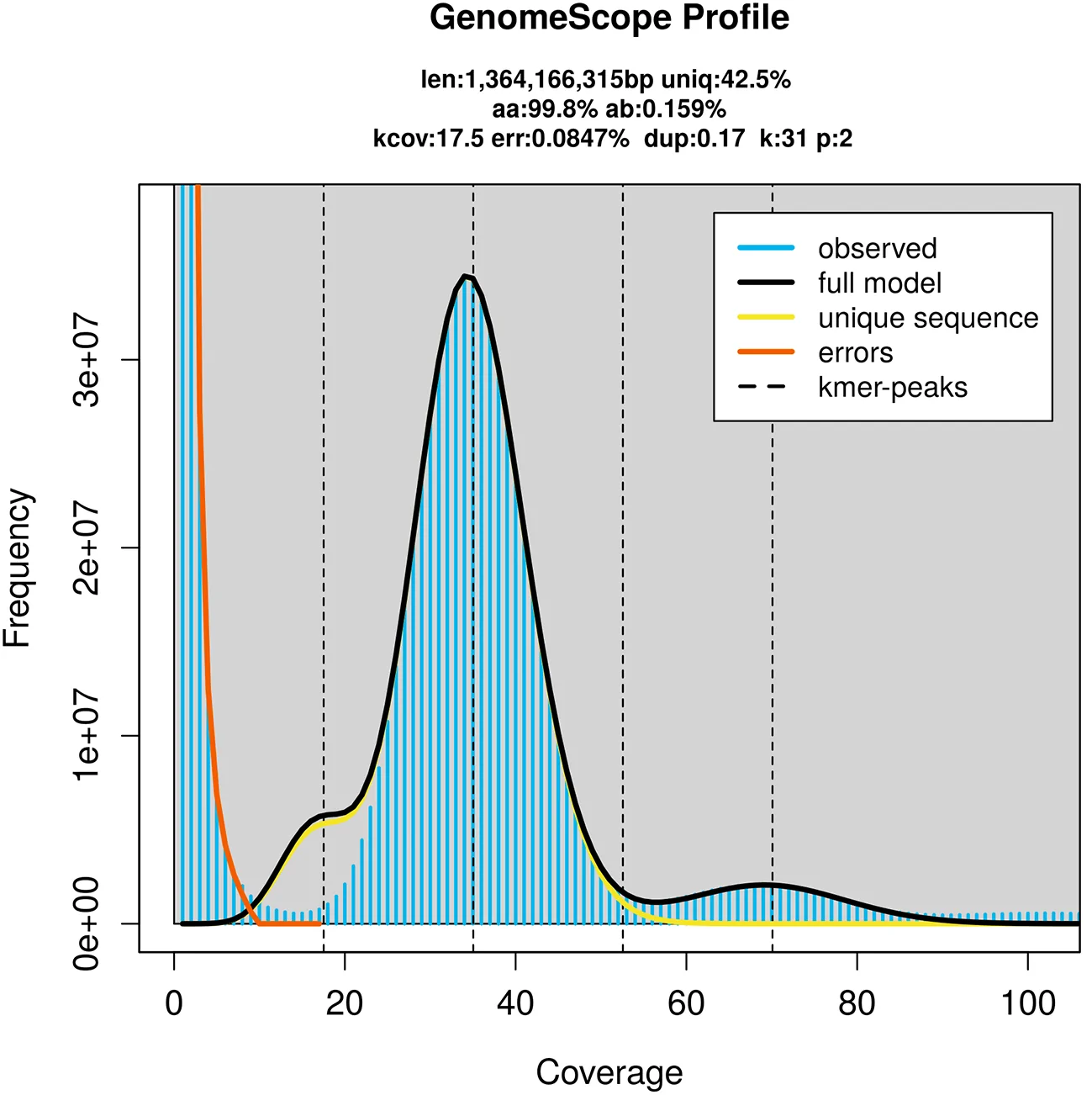
Frequency distribution of *k*-mers generated using GenomeScope2. The plot shows observed and modelled *k*-mer spectra, providing estimates of genome size, heterozygosity, and repeat content based on unassembled sequencing reads.

**Table 1.** Specimen and sequencing data for *Moehringia trinervia* (BioProject PRJEB74623).

Platform	PacBio HiFi	Hi-C	RNA-seq
**ToLID**	dcMoeTrin1	dcMoeTrin1	dcMoeTrin1
**Specimen ID**	EDTOL05488	EDTOL05488	EDTOL05488
**BioSample (source individual)**	SAMEA114214469	SAMEA114214469	SAMEA114214469
**BioSample (tissue)**	SAMEA114214548	SAMEA114214548	SAMEA114214548
**Tissue**	whole plant	whole plant	whole plant
**Instrument**	Revio	Illumina NovaSeq X	Illumina NovaSeq X
**Run accessions**	ERR12875171	ERR12893012	ERR12893013
**Read count total**	3.91 million reads	690.14 million read pairs	34.26 million read pairs
**Base count total**	49.19 Gb	208.42 Gb	10.35 Gb

### Assembly statistics

The genome was assembled into two haplotypes using Hi-C phasing. Haplotype 1 was curated to chromosome level, while haplotype 2 was assembled to scaffold level. The final assembly has a total length of 1 244.27 Mb in 252 scaffolds, with 202 gaps, and a scaffold N50 of 103.01 Mb ( Table 2).

**Table 2.** Genome assembly statistics for *Moehringia trinervia.*

Genome assembly	Haplotype 1	Haplotype 2
**Assembly name**	dcMoeTrin1.hap1.1	dcMoeTrin1.hap2.1
**Assembly accession**	GCA_964106765.1	GCA_964106845.1
**Assembly level**	chromosome	scaffold
**Span (Mb)**	1 244.27	1 242.42
**Number of chromosomes**	12	scaffold-level
**Number of contigs**	454	407
**Contig N50**	10.55 Mb	10.36 Mb
**Number of scaffolds**	252	212
**Scaffold N50**	103.01 Mb	104.09 Mb
**Longest scaffold length (Mb)**	120.32	202.29
**Organelles**	Mitochondrial genome: 335.44 kb; Plastid genome: 149.69 kb	-

Most of the assembly sequence (98.82%) was assigned to 12 chromosomal-level scaffolds. These chromosome-level scaffolds, confirmed by Hi-C data, are named according to size ( Figure 3; Table 3).

**Figure 3.**
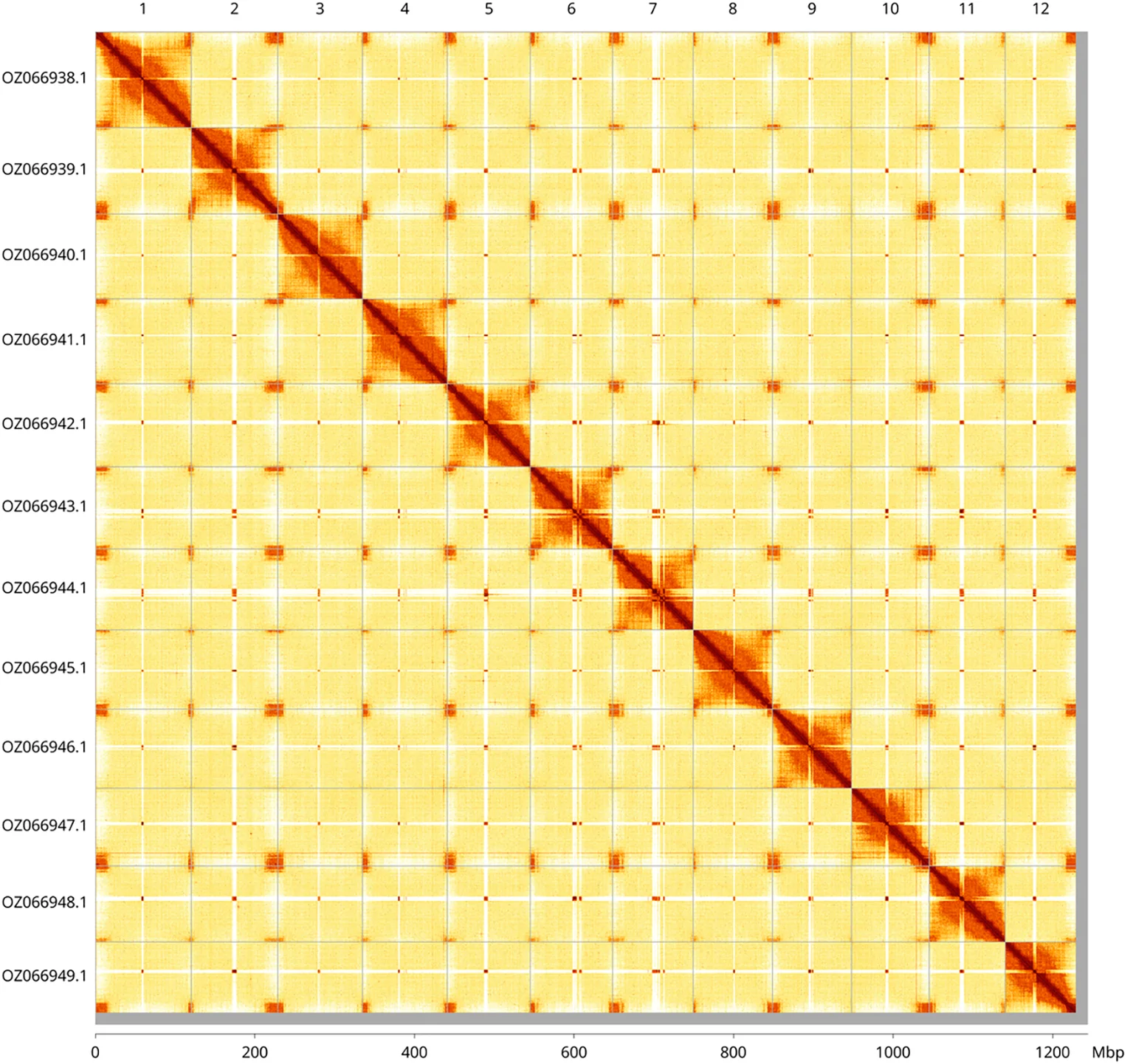
Hi-C contact map of the *Moehringia trinervia* genome assembly. Assembled chromosomes are shown in order of size and labelled along the axes, with a megabase scale shown below. The plot was generated using PretextSnapshot.

**Table 3.** Chromosomal pseudomolecules in the haplotype 1 genome assembly of *Moehringia trinervia* dcMoeTrin1.

INSDC accession	Molecule	Length (Mb)	GC%
OZ066938.1	1	120.32	36.50
OZ066939.1	2	108.34	36
OZ066940.1	3	106.45	36.50
OZ066941.1	4	106.30	36.50
OZ066942.1	5	104.07	36.50
OZ066943.1	6	103.01	36
OZ066944.1	7	101.04	35.50
OZ066945.1	8	99.45	36.50
OZ066946.1	9	99.10	36.50
OZ066947.1	10	97.19	36.50
OZ066948.1	11	95.31	36
OZ066949.1	12	89.02	36

The mitochondrial genome (length 335.44 kb, OZ066950.1) and plastid genome (length 149.69 kb, OZ066951.1) were also assembled. These sequences are included as contigs in the multifasta file of the genome submission and as standalone records.

### Assembly quality metrics

For haplotype 1, the estimated QV is 65.0, and for haplotype 2, 65.3. When the two haplotypes are combined, the assembly achieves an estimated QV of 65.1. The *k*-mer completeness is 99.30% for haplotype 1, 99.27% for haplotype 2, and 99.32% for the combined haplotypes ( Figure 4).

**Figure 4.**
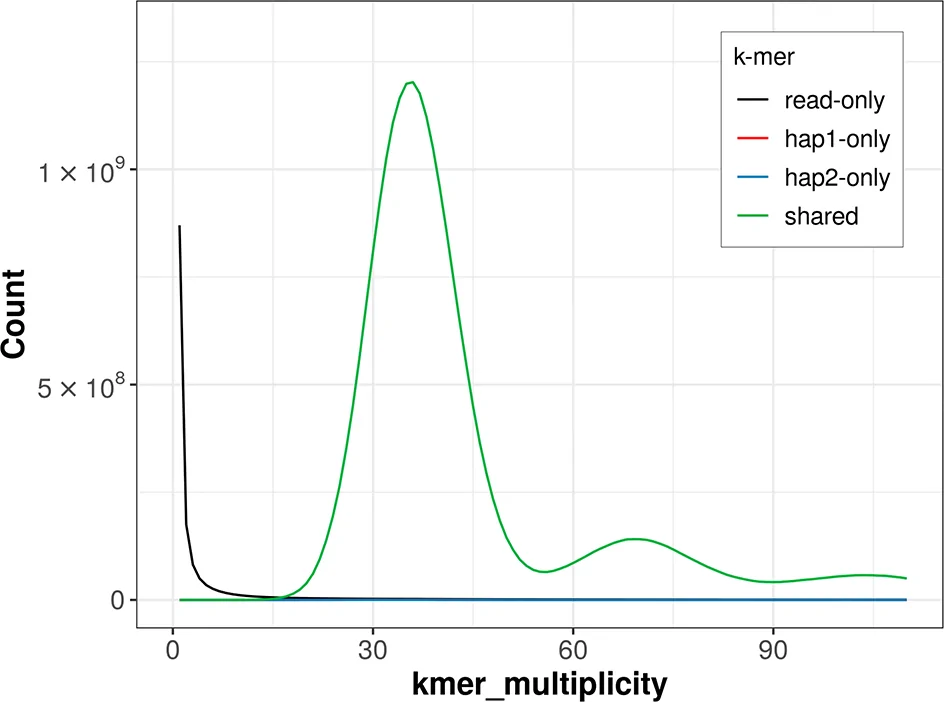
Evaluation of *k*-mer completeness using MerquryFK. This plot illustrates the recovery of *k*-mers from the original read data in the final assemblies. The horizontal axis represents *k*-mer multiplicity, and the vertical axis shows the number of *k*-mers. The black curve represents *k*-mers that appear in the reads but are not assembled. The green curve corresponds to *k*-mers shared by both haplotypes, and the red and blue curves show *k*-mers found only in one of the haplotypes.

BUSCO analysis using the eudicots_odb10 reference set ( *n* = 2 326) identified 96.0% of the expected gene set (single = 88.5%, duplicated = 7.5%) for haplotype 1. The snail plot in Figure 5 summarises the scaffold length distribution and other assembly statistics for haplotype 1. The blob plot in Figure 6 shows the distribution of scaffolds by GC proportion and coverage for haplotype 1.

**Figure 5.**
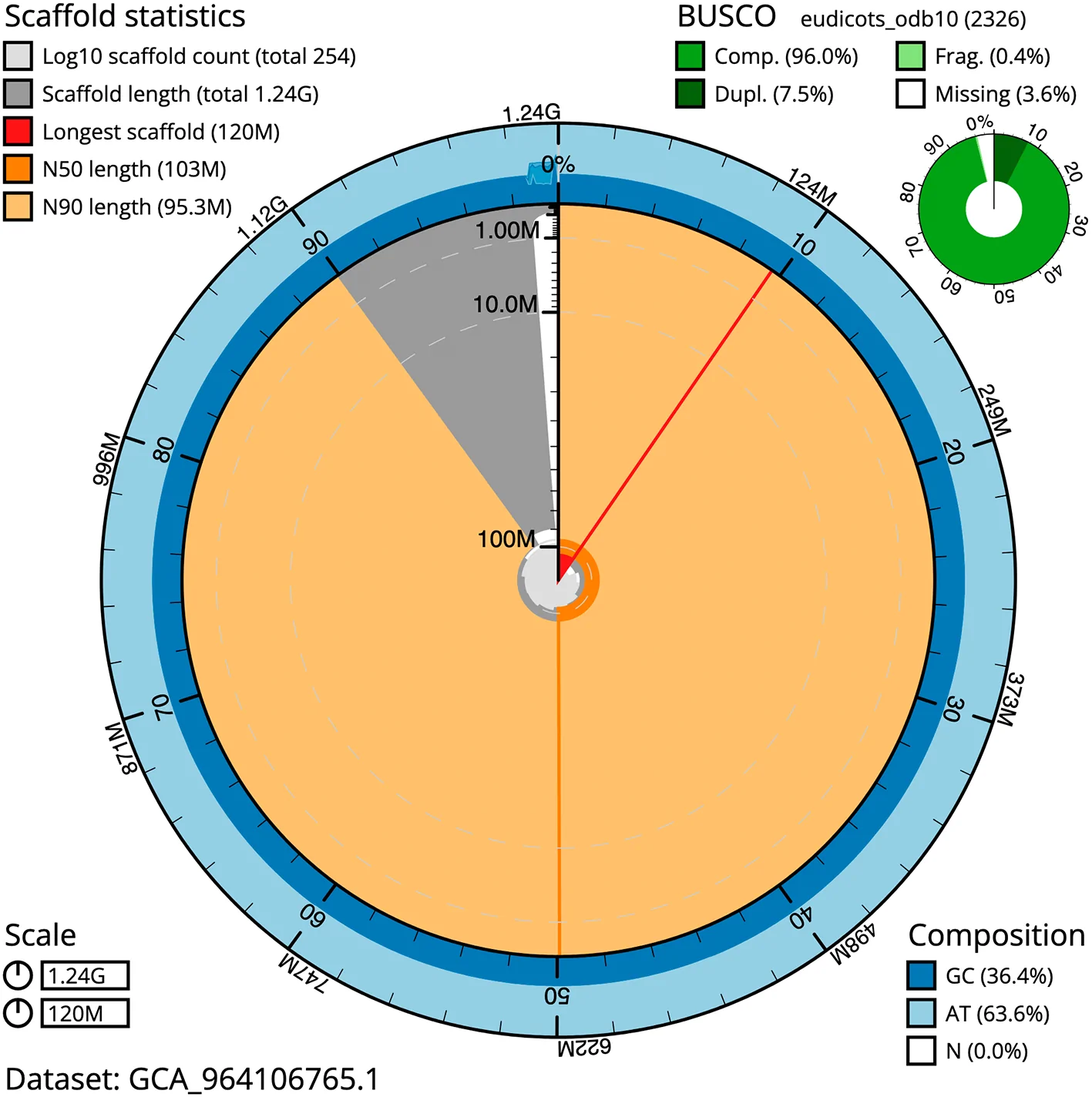
Assembly metrics for dcMoeTrin1.hap1.1. The BlobToolKit snail plot provides an overview of assembly metrics and BUSCO gene completeness. The circumference represents the length of the whole genome sequence, and the main plot is divided into 1,000 bins around the circumference. The outermost blue tracks display the distribution of GC, AT, and N percentages across the bins. Scaffolds are arranged clockwise from longest to shortest and are depicted in dark grey. The longest scaffold is indicated by the red arc, and the deeper orange and pale orange arcs represent the N50 and N90 lengths. A light grey spiral at the centre shows the cumulative scaffold count on a logarithmic scale. A summary of complete, fragmented, duplicated, and missing BUSCO genes in the set is presented at the top right. An interactive version of this figure can be accessed on the BlobToolKit viewer.

**Figure 6.**
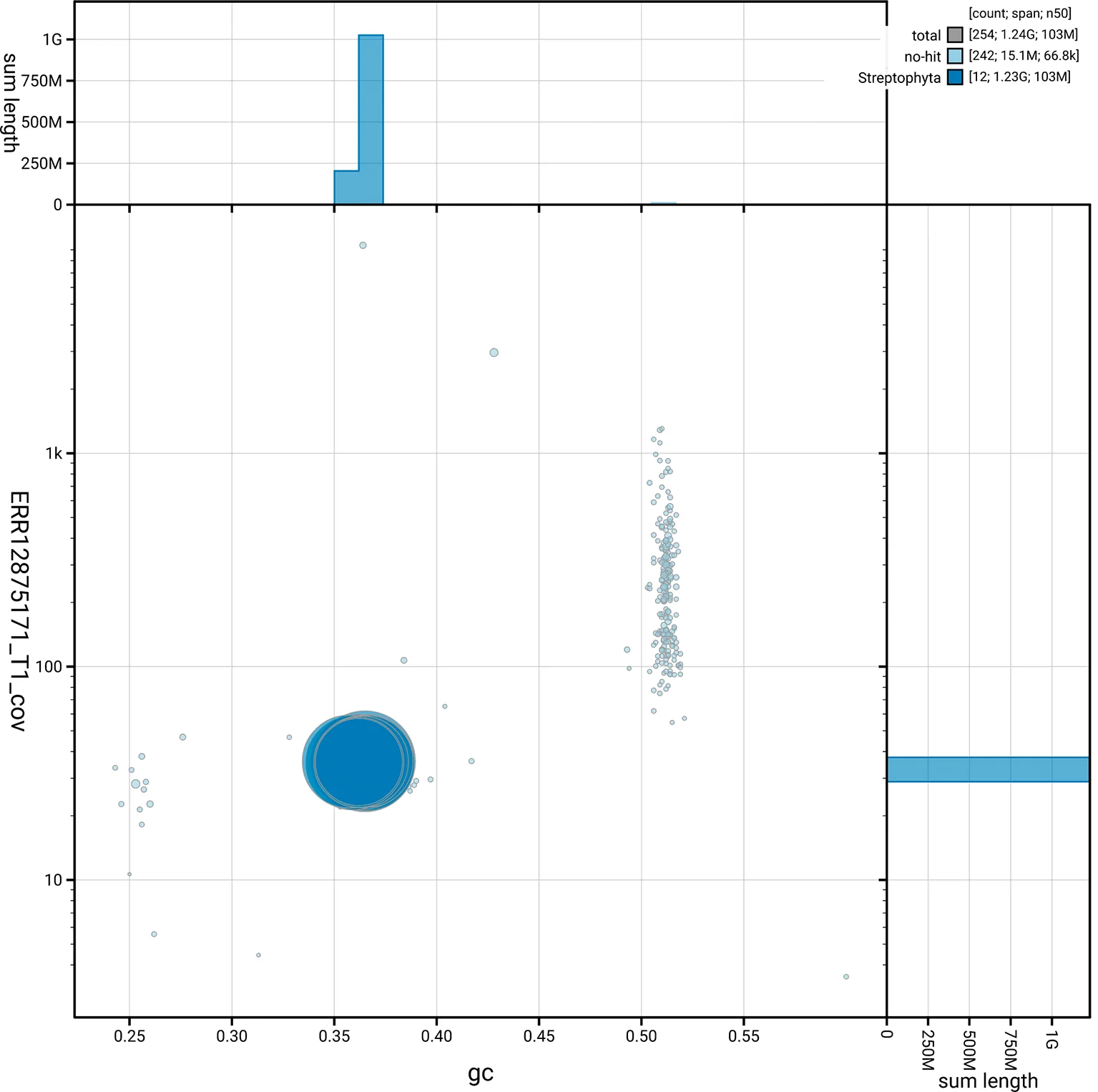
BlobToolKit blob plot for dcMoeTrin1.hap1.1. The plot shows base coverage (vertical axis) and GC content (horizontal axis). The circles represent scaffolds, with the size proportional to scaffold length and the colour representing phylum membership. The histograms along the axes display the total length of sequences distributed across different levels of coverage and GC content. An interactive version of this figure is available on the BlobToolKit viewer.

Table 4 lists the assembly metric benchmarks adapted from Rhie *et al.* (2021) and the Earth BioGenome Project Report on Assembly Standards January 2026. The EBP metric calculated for the haplotype 1 is **7.C.Q65**, meeting the recommended 6.C.Q40 reference standard.

**Table 4.** Earth biogenome project summary metrics for the *Moehringia trinervia* assembly.

Measure	Value	Benchmark
EBP summary (haplotype 1)	7.C.Q65	6.C.Q40
Contig N50 length	10.55 Mb	≥ 1 Mb
Scaffold N50 length	103.01 Mb	= chromosome N50
Consensus quality (QV)	Haplotype 1: 65.0; haplotype 2: 65.3; combined: 65.1	≥ 40
*k*-mer completeness	Haplotype 1: 99.30%; Haplotype 2: 99.27%; combined: 99.32%	≥ 95%
BUSCO	C:96.0% [S:88.5%, D:7.5%], F:0.4%, M:3.6%, n:2 326	S > 90%; D < 5%
Percentage of assembly assigned to chromosomes	98.82%	≥ 90%

**Table 5.** Software versions and sources used for *Moehringia trinervia.*

Software	Version	Source
BLAST	2.14.0	ftp://ftp.ncbi.nlm.nih.gov/blast/executables/blast+/
BlobToolKit	4.3.9	https://github.com/blobtoolkit/blobtoolkit
BUSCO	5.5.0	https://gitlab.com/ezlab/busco
bwa-mem2	2.2.1	https://github.com/bwa-mem2/bwa-mem2
DIAMOND	2.1.8	https://github.com/bbuchfink/diamond
fasta_windows	0.2.4	https://github.com/tolkit/fasta_windows
FastK	1.1	https://github.com/thegenemyers/FASTK
GenomeScope2.0	2.0.1	https://github.com/tbenavi1/genomescope2.0
Gfastats	1.3.6	https://github.com/vgl-hub/gfastats
Hifiasm	0.19.8-r603	https://github.com/chhylp123/hifiasm
HiGlass	1.13.4	https://github.com/higlass/higlass
MerquryFK	1.1.2	https://github.com/thegenemyers/MERQURY.FK
Minimap2	2.24-r1122	https://github.com/lh3/minimap2
Oatk	0.9	https://github.com/c-zhou/oatk
MultiQC	1.14; 1.17 and 1.18	https://github.com/MultiQC/MultiQC
Nextflow	23.10.0	https://github.com/nextflow-io/nextflow
PretextSnapshot	0.0.5	https://github.com/sanger-tol/PretextSnapshot
PretextView	1.0.3	https://github.com/sanger-tol/PretextView
samtools	1.19.2	https://github.com/samtools/samtools
sanger-tol/ascc	0.1.0	https://github.com/sanger-tol/ascc
sanger-tol/blobtoolkit	0.6.0	https://github.com/sanger-tol/blobtoolkit
sanger-tol/curationpretext	1.4.2	https://github.com/sanger-tol/curationpretext
Seqtk	1.3	https://github.com/lh3/seqtk
Singularity	3.9.0	https://github.com/sylabs/singularity
TreeVal	1.4.0	https://github.com/sanger-tol/treeval
YaHS	1.2a.2	https://github.com/c-zhou/yahs

**Notes:** The EBP summary uses log10(Contig N50); chromosome-level (C) or log10(Scaffold N50); Q (Merqury QV). BUSCO: C = complete; S = single-copy; D = duplicated; F = fragmented; M = missing; n = orthologues

### Genome annotation report

The *Moehringia trinervia* genome assembly (GCA_964106765.1) was annotated by Ensembl at the European Bioinformatics Institute (EBI). This annotation includes 54 746 transcribed mRNAs from 21 033 protein-coding and 28 076 non-coding genes. The average transcript length is 2 892.55 bp, with an average of 1.11 coding transcripts per gene and 3.64 exons per transcript. For further information about the annotation, please refer to the annotation page on Ensembl.

## Author information

• Members of the Royal Botanic Garden Edinburgh Genome Acquisition Lab
• Members of the Plant Genome Sizing Collective
• Members of the Darwin Tree of Life Barcoding collective
• Members of the Wellcome Sanger Institute Tree of Life Management, Samples and Laboratory team
• Members of Wellcome Sanger Institute Scientific Operations – Sequencing Operations
• Members of the Wellcome Sanger Institute Tree of Life Core Informatics team
• Members of the Tree of Life Core Informatics collective
• Members of the Darwin Tree of Life Consortium

## Wellcome Sanger Institute – Legal and Governance

The materials that have contributed to this genome note have been supplied by a Darwin Tree of Life Partner. The submission of materials by a Darwin Tree of Life Partner is subject to the **‘Darwin Tree of Life Project Sampling Code of Practice’**, which can be found in full on the Darwin Tree of Life website. By agreeing with and signing up to the Sampling Code of Practice, the Darwin Tree of Life Partner agrees they will meet the legal and ethical requirements and standards set out within this document in respect of all samples acquired for, and supplied to, the Darwin Tree of Life Project. Further, the Wellcome Sanger Institute employs a process whereby due diligence is carried out proportionate to the nature of the materials themselves, and the circumstances under which they have been/are to be collected and provided for use. The purpose of this is to address and mitigate any potential legal and/or ethical implications of receipt and use of the materials as part of the research project, and to ensure that in doing so we align with best practice wherever possible. The overarching areas of consideration are:

• Ethical review of provenance and sourcing of the material
• Legality of collection, transfer and use (national and international)

Each transfer of samples is further undertaken according to a Research Collaboration Agreement or Material Transfer Agreement entered into by the Darwin Tree of Life Partner, Genome Research Limited (operating as the Wellcome Sanger Institute), and in some circumstances, other Darwin Tree of Life collaborators.

## Data Availability

European Nucleotide Archive: Moehringia trinervia. Accession number PRJEB74623; https://identifiers.org/ena.embl/PRJEB74623. The genome sequence is released openly for reuse. The *Moehringia trinervia* genome sequencing initiative is part of the Darwin Tree of Life Project (PRJEB40665) and the Sanger Institute Tree of Life Programme (PRJEB43745). All raw sequence data and the assembly have been deposited in INSDC databases. Raw data and assembly accession identifiers are reported in Tables 1 and 2. Pipelines used for genome assembly at the WSI Tree of Life are available at https://pipelines.tol.sanger.ac.uk/pipelines. Table 5 lists software versions used in this study.

## References

[ref1] Altschul SF Gish W Miller W : Basic Local Alignment Search Tool. *Journal of Molecular Biology.* 1990;215(3):403–410. 2231712 10.1016/S0022-2836(05)80360-2

[ref2] Bateman A Martin M-J Orchard S : UniProt: The Universal Protein Knowledgebase in 2023. *Nucleic Acids Res.* 2023;51(D1):D523–D531. 36408920 10.1093/nar/gkac1052 PMC9825514

[ref3] Buchfink B Reuter K Drost H-G : Sensitive protein alignments at tree-of-life scale using DIAMOND. *Nat Methods.* 2021;18(4):366–368. 33828273 10.1038/s41592-021-01101-x PMC8026399

[ref4] Casazza G Borghesi B Roccotiello E : Dispersal mechanisms in some representatives of the genus *Moehringia* L. (Caryophyllaceae). *Acta Oecologica.* 2008;33(2):246–252. 10.1016/j.actao.2007.11.003

[ref5] Challis R Richards E Rajan J : BlobToolKit – interactive quality assessment of genome assemblies. *G3 Genes|Genomes|Genetics.* 2020;10(4):1361–1374. 32071071 10.1534/g3.119.400908 PMC7144090

[ref6] Chase MW Hills HH : Silica gel: An ideal material for field preservation of leaf samples for DNA studies. *Taxon.* 1991;40(2):215–220. 10.2307/1222975

[ref7] Cheng H Concepcion GT Feng X : Haplotype-resolved *de novo* assembly using phased assembly graphs with Hifiasm. *Nat Methods.* 2021;18(2):170–175. 33526886 10.1038/s41592-020-01056-5 PMC7961889

[ref8] Cheng H Jarvis ED Fedrigo O : Haplotype-resolved assembly of diploid genomes without parental data. *Nat Biotechnol.* 2022;40(9):1332–1335. 35332338 10.1038/s41587-022-01261-x PMC9464699

[ref9] Danecek P Bonfield JK Liddle J : Twelve years of SAMtools and BCFtools. *GigaScience.* 2021;10(2):giab008. 33590861 10.1093/gigascience/giab008 PMC7931819

[ref10] Doležel J Greilhuber J Suda J : Estimation of nuclear DNA content in plants using flow cytometry. *Nat Protoc.* 2007;2(9):2233–2244. 17853881 10.1038/nprot.2007.310

[ref11] Ewels P Magnusson M Lundin S : MultiQC: Summarize analysis results for multiple tools and samples in a single report. *Bioinformatics.* 2016;32(19):3047–3048. 27312411 10.1093/bioinformatics/btw354 PMC5039924

[ref12] Ewels PA Peltzer A Fillinger S : The nf-core framework for community-curated bioinformatics pipelines. *Nature Biotechnology.* 2020;38(3):276–278. 32055031 10.1038/s41587-020-0439-x

[ref13] Formenti G Abueg L Brajuka A : Gfastats: Conversion, evaluation and manipulation of genome sequences using assembly graphs. *Bioinformatics.* 2022;38(17):4214–4216. 35799367 10.1093/bioinformatics/btac460 PMC9438950

[ref14] Godzik B : *Moehringia trinervia* (L.) Clairv. – a bioindicator of heavy metal pollution of the environment. *Acta Soc Bot Pol.* 1992;61(3–4):409–417. 10.5586/asbp.1992.036

[ref15] Henniges MC Powell RF Mian S : A taxonomic, genetic and ecological data resource for the vascular plants of Britain and Ireland. *Sci Data.* 2022;9(1):1. 35013360 10.1038/s41597-021-01104-5 PMC8748506

[ref16] Howard C Denton A Jackson B : On the path to reference genomes for all biodiversity: Lessons learned and laboratory protocols created in the Sanger Tree of Life core laboratory over the first 2000 species. *bioRxiv.* 2025. 10.1101/2025.04.11.648334 PMC12548527 41129326

[ref17] Howe K Chow W Collins J : Significantly improving the quality of genome assemblies through curation. *GigaScience.* 2021;10(1):giaa153. 33420778 10.1093/gigascience/giaa153 PMC7794651

[ref18] Kerpedjiev P Abdennur N Lekschas F : HiGlass: Web-based visual exploration and analysis of genome interaction maps. *Genome Biol.* 2018;19(1):125. 30143029 10.1186/s13059-018-1486-1 PMC6109259

[ref19] Kurtzer GM Sochat V Bauer MW : Singularity: Scientific containers for mobility of compute. *PLoS One.* 2017;12(5):e0177459. 28494014 10.1371/journal.pone.0177459 PMC5426675

[ref20] Li H : Minimap2: Pairwise alignment for nucleotide sequences. *Bioinformatics.* 2018;34(18):3094–3100. 29750242 10.1093/bioinformatics/bty191 PMC6137996

[ref21] Loureiro J Rodriguez E Doležel J : Two new nuclear isolation buffers for plant DNA flow cytometry: A test with 37 species. *Ann Bot.* 2007;100(4):875–888. 17684025 10.1093/aob/mcm152 PMC2749623

[ref22] Lövkvist B Hultgård U-M : *Chromosome Numbers in South Swedish Vascular Plants.* Opera Botanica;1999; vol.137.

[ref23] Manni M Berkeley MR Seppey M : BUSCO update: Novel and streamlined workflows along with broader and deeper phylogenetic coverage for scoring of eukaryotic, prokaryotic, and viral genomes. *Mol Biol Evol.* 2021;38(10):4647–4654. 34320186 10.1093/molbev/msab199 PMC8476166

[ref24] Merkel D : Docker: Lightweight Linux containers for consistent development and deployment. *Linux J.* 2014;2014(239):2. 10.5555/2600239.2600241

[ref25] O’Leary NA Cox E Holmes JB : Exploring and retrieving sequence and metadata for species across the tree of life with NCBI Datasets. *Sci Data.* 2024;11(1):732. 38969627 10.1038/s41597-024-03571-y PMC11226681

[ref26] Pellicer J Powell RF Leitch IJ : The application of flow cytometry for estimating genome size, ploidy level endopolyploidy, and reproductive modes in plants. Besse P , editor. *Methods in Molecular Biology.* New York, NY:2021;2222:325–61. 33301101 10.1007/978-1-0716-0997-2_17

[ref27] Ranallo-Benavidez TR Jaron KS Schatz MC : GenomeScope 2.0 and Smudgeplot for reference-free profiling of polyploid genomes. *Nat Commun.* 2020;11(1):1432. 32188846 10.1038/s41467-020-14998-3 PMC7080791

[ref28] Rao SSP Huntley MH Durand NC : A 3D map of the human genome at kilobase resolution reveals principles of chromatin looping. *Cell.* 2014;159(7):1665–1680. 25497547 10.1016/j.cell.2014.11.021 PMC5635824

[ref29] Ratnasingham S Hebert PDN : BOLD: The Barcode of Life Data System (http://www.barcodinglife.org). *Mol Ecol Notes.* 2007;7(3):355–364. 18784790 10.1111/j.1471-8286.2007.01678.x PMC1890991

[ref30] Rhie A McCarthy SA Fedrigo O : Towards complete and error-free genome assemblies of all vertebrate species. *Nature.* 2021;592(7856):737–746. 33911273 10.1038/s41586-021-03451-0 PMC8081667

[ref31] Rhie A Walenz BP Koren S : Merqury: Reference-free quality, completeness, and phasing assessment for genome assemblies. *Genome Biol.* 2020;21(1):245. 32928274 10.1186/s13059-020-02134-9 PMC7488777

[ref32] Stace CA : *New Flora of the British Isles.* C&M Floristics;2019.

[ref33] Twyford AD Beasley J Barnes I : A DNA barcoding framework for taxonomic verification in the Darwin Tree of Life Project. *Wellcome Open Res.* 2024;9:339. 39386966 10.12688/wellcomeopenres.21143.1 PMC11462125

[ref34] Vasimuddin M Misra S Li H : Efficient architecture-aware acceleration of BWA-MEM for multicore systems. *2019 IEEE International Parallel and Distributed Processing Symposium (IPDPS).* IEEE;2019;314–24. 10.1109/IPDPS.2019.00041

[ref35] Zhou C Brown M Blaxter M : Oatk: A de novo assembly tool for complex plant organelle genomes. *Genome Biology.* 2025;26(1):235. 40775726 10.1186/s13059-025-03676-6 PMC12329965

[ref36] Zhou C McCarthy SA Durbin R : YaHS: Yet another Hi-C scaffolding tool. *Bioinformatics.* 2023;39(1):btac808. 36525368 10.1093/bioinformatics/btac808 PMC9848053

