# Supplementary material for: The genome sequence of Moehringia trinervia (L.) Clairv., 1811 (Caryophyllales: Caryophyllaceae)
Source: Wellcome Open Res. 2026 Jun 13;11:360. [Version 1] doi: 10.12688/wellcomeopenres.26721.1 (PMC13673086; doi:10.12688/wellcomeopenres.26721.1)
Supplement: Peer review history

# Reviewer report: Reviewer response for version 1

**DOI:** 10.21956/wellcomeopenres.29423.r164546

**Published:** 2026-10-15

**Authors:** W. Brad Barbazuk

**Affiliations:** 1 University of Florida, Gainesville, Florida, USA

This report describes the genome assembly of *Moehringia trinervia*   ( *M. trinervia* ;  Three-nerved Sandwort; Streptophyta; Magnoliopsida; Caryophyllales; Caryophyllaceae).  This plant has a broad distribution across Europe and Asia, and is common to the British Isles. As it is native to Britain and Ireland, this species is a natural addition to the Darwin Tree of Life project, and it’s genome sequence was determined as part of this project. Additionally, *M. trinervia* has been identified as a bioindicator of heavy metal pollution and accumulates elevated levels of heavy metals when grown in contaminated soils, thus making it a useful indicator for assessing anthropogenic impacts on forest floor vegetation.

 The methodology, sequence and assembly, HiC integration, scaffolding, and QC integration use well-established, state-of-the -art techniques. The computational processes use well-established tools and workflows optimized for this type of work. In general, the assembly and annotation are very high quality and will support the botany and plant science communities.

 I have the following questions, which should be addressed by the authors.

1. The manuscript points out repeat content, as determined by K-mer-based methods. Does the  Ensemble annotation workflow include repeat identification and classification? If so, these may be useful data for the community.  Assuming the Ensemble pipeline does handle repeat identification, are the repeat annotations included in the final GFF/GTF annotation files?
2. Regarding the sequences within the final assembly that were identified as ‘contaminant’ by the ASCC pipeline. How were these removed? Is this automated within the pipeline, or manually reviewed and edited out?
3. I find the summary in the snail plot far more interpretable than the plot itself.  Is there not a better visual to use in place of the snail plot? I, personally, find these hard to interpret, but that may simply be a deficiency on my part.
4. Haplotype 1 is finished at the chromosome level, while haplotype 2 is scaffolded.  While sequence variation can be determined by comparing the scaffolded Haplotype 2 to the finished Haplotype I, there may be interesting and important rearrangements and other structural variation differences between haplotypes which may not be identified without finished versions of both. Were there any indications from the re-alignment of the PacBio reads to haplotype 2 that there were potential rearrangements or SV? What time and effort would it take to finish haplotype 2 with the well-established workflows and pipelines in use at the Sanger ?
5. Was the depth of HiFi reads sufficient to not require subsequent base-by-base polishing with Illumina reads?
6. Illumina RNA-Seq was used to help guide the annotation process. The manuscript suggests that whole-plant tissue was used and that ~38M read pairs were obtained. What was the rationale for 38M? Is whole plant the best option, or would a selection of major tissues potentially provide a more comprehensive annotation? Finally, why does the process not include some IsoSeq or nanopore RNA-SEQ reads to aid in gene assembly?

Are sufficient details of methods and materials provided to allow replication by others?

Yes

Is the rationale for creating the dataset(s) clearly described?

Yes

Are the datasets clearly presented in a useable and accessible format?

Yes

Are the protocols appropriate and is the work technically sound?

Yes

Reviewer Expertise:

Bioinformatics, comparative genomics, genetics

I confirm that I have read this submission and believe that I have an appropriate level of expertise to confirm that it is of an acceptable scientific standard.

# Reviewer report: Reviewer response for version 1

**DOI:** 10.21956/wellcomeopenres.29423.r160321

**Published:** 2026-08-05

**Authors:** Pawan Kumar Jayaswal

**Affiliations:** 1 Bihar Agricultural University, Sabour, Bihar, India

The methodology has been clearly described following the Darwin Tree of Life Project in the manuscript entitled *"The genome sequence of Moehringia trinervia (L.) Clairv., 1811 (Caryophyllales: Caryophyllaceae)"* . The manuscript presents a high-quality genome assembly and associated datasets. It is recommended for publication after the authors address the following comments:

1. In the haplotype-resolved genome assembly of *Moehringia trinervia* , there is an inconsistency in the reported scaffold counts. Table 2 reports 252 scaffolds for Haplotype 1, whereas the snail plot in Figure 5 indicates 254 scaffolds. Please reconcile this discrepancy or clarify whether the figure includes the two organellar contigs.
2. Table 1 reports 10.35 Gb of RNA-seq data comprising 34.26 million read pairs. However, the manuscript does not explicitly state whether these RNA-seq data were used to support the Ensembl genome annotation. Please clarify their role in the annotation pipeline.
3. Haplotype 1 was assembled to chromosome level across 12 pseudomolecules spanning 1,244.27 Mb, with a reported repeat content of 57.54%. However, the manuscript does not provide a detailed characterization of the repeat annotation. Please include a comprehensive description of the repeat annotation results.

Are sufficient details of methods and materials provided to allow replication by others?

Yes

Is the rationale for creating the dataset(s) clearly described?

Yes

Are the datasets clearly presented in a useable and accessible format?

Yes

Are the protocols appropriate and is the work technically sound?

Yes

Reviewer Expertise:

Functional genomics

I confirm that I have read this submission and believe that I have an appropriate level of expertise to confirm that it is of an acceptable scientific standard.
